# Stereotyped initiation of retinal waves by bipolar cells via presynaptic NMDA autoreceptors

**DOI:** 10.1038/ncomms12650

**Published:** 2016-09-02

**Authors:** Rong-wei Zhang, Xiao-quan Li, Koichi Kawakami, Jiu-lin Du

**Affiliations:** 1 Institute of Neuroscience, State Key Laboratory of Neuroscience, Center for Excellence in Brain Science and Intelligence Technology, Shanghai Institutes for Biological Sciences, Chinese Academy of Sciences, 320 Yue-Yang Road, Shanghai 200031, China; 2 Division of Molecular and Developmental Biology, National Institute of Genetics, 1111 Yata, Mishima, Shizuoka 411-8540, Japan; 3 School of Life Science and Technology, ShanghaiTech University, 319 Yue-Yang Road, Shanghai 200031, China; 4 University of Chinese Academy of Sciences, 19A Yu-Quan Road, Beijing 100049, China

## Abstract

Glutamatergic retinal waves, the spontaneous patterned neural activities propagating among developing retinal ganglion cells (RGCs), instruct the activity-dependent refinement of visuotopic maps. However, its initiation and underlying mechanism remain largely elusive. Here using larval zebrafish and multiple *in vivo* approaches, we discover that bipolar cells (BCs) are responsible for the generation of glutamatergic retinal waves. The wave originates from BC axon terminals (ATs) and propagates laterally to nearby BCs and vertically to downstream RGCs and the optic tectum. Its initiation is triggered by the activation of and consequent glutamate release from BC ATs, and is mediated by the *N*-methyl-D-aspartate subtype of glutamate receptors (NMDARs) expressed at these ATs. Intercellular asymmetry of NMDAR expression at BC ATs enables the preferential initiation of waves at the temporal retina, where BC ATs express more NMDARs. Thus, our findings indicate that glutamatergic retinal waves are initiated by BCs through a presynaptic NMDA autoreceptor-dependent process.

## Abstract (web summary)

Retinal waves are important for visual system development. However, the mechanism involved in their generation remains largely unknown. Here using *in vivo* two-photon imaging the authors identify the presence of retinal waves in zebrafish larvae and find that they are initiated at bipolar cells via presynaptic NMDARs.

## Graphical abstract

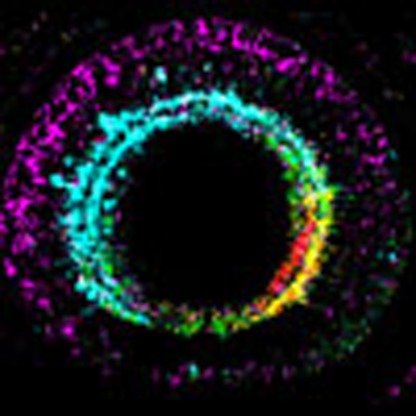

During early development, the retina exhibits spontaneous patterned neural activities sweeping in a wave-like manner across neighbouring retinal ganglion cells (RGCs), termed retinal waves^1^,^2^,^3^. The retinal wave has been observed in many vertebrate species, including reptiles, birds, rodents, rabbits, ferrets and non-human primates^3^,^4^,^5^. It can propagate via the optic nerve to the central regions of the visual system, including the dorsal lateral geniculate nucleus, superior colliculus and primary visual cortex^6^,^7^,^8^, and plays a crucial role in the activity-dependent refinement of visual topographic maps^3^,^4^,^5^,^9^,^10^,^11^,^12^.

With the maturation of retinal circuitries, the excitatory drive of retinal waves switches from cholinergic to glutamatergic^13^,^14^,^15^. It has been known that cholinergic retinal waves are initiated by starburst amacrine cells and mediated by nicotinic acetylcholine receptors^16^,^17^,^18^. However, mechanisms underlying the generation of glutamatergic retinal waves are not fully understood yet. Previous studies showed that glutamatergic retinal waves could be abolished by blockade of glutamatergic synaptic transmission^15^,^19^,^20^,^21^. Their propagation at the ganglion cell layer was accompanied with extrasynaptic glutamate spillover at the inner plexiform layer (IPL)^20^,^22^. A recent electrophysiological study showed that bipolar cells (BCs) and RGCs exhibited correlated spontaneous depolarization in isolated immature mouse retinae^23^. These studies suggest that BCs may provide excitatory inputs to RGCs to generate glutamatergic waves. However, it is still unclear whether and how BCs initiate glutamatergic retinal waves^24^.

Taking advantage of the optical transparency and small size of the larval zebrafish's retina and the availability of genetic tools, we examined the role of BCs in the generation of glutamatergic retinal waves by combining multiple *in vivo* methods, including two-photon calcium imaging, whole-cell patch-clamp recording, optogenetics, glutamate uncaging and glutamate imaging. During a narrow developmental window from 2.5 to 3.5 days post-fertilization (d.p.f.), BCs in intact zebrafish larvae exhibited spontaneous wave-like neuronal activities, which originated from BC axon terminals (ATs) and then swept across the population of BCs. Channelrhodopsin-2 (ChR2)-based optogenetic or local puffing-based pharmacological activation of a small cluster of BC ATs efficiently triggered wave activities in BC populations. The generation of BC waves was dependent on glutamate release from BC ATs, because it was prevented or facilitated respectively by blockade or elevation of glutamate release, and was accompanied with wave-like spontaneous spillover of extracellular glutamate in the inner plexiform layer (IPL). Furthermore, BC ATs expressed the *N*-methyl-D-aspartate subtype of glutamate receptors (NMDARs), and the activation of these receptors was necessary and sufficient for wave generation. In addition, the existence of gap junctions between BCs was necessary for the wave generation and propagation. Importantly, waves in RGCs were correlated with those in BCs and the optic tectum (OT), and could be induced by optogenetic activation of BC ATs, suggesting that wave activities can propagate from BCs to RGCs and even to the higher visual center. Contrary to the random initiation of retinal waves reported in previous in vitro studies^3^,^4^,^25^, we found that the waves in BCs and RGCs started preferentially at the temporal retina, and waves in the OT started at the anterior neuropil, the topographical recipient of temporal RGC axons. This stereotyped initiation pattern was mainly because BC ATs at the temporal retina expressed a high density of functional NMDARs and thus possessed a relatively low threshold for wave generation. Taken together, our results elucidate that the ATs of BCs initiate glutamatergic retinal waves through a NMDA autoreceptor-dependent mechanism.

## Results

### Developing bipolar cells exhibit spontaneous retinal waves

**Figure 1:**
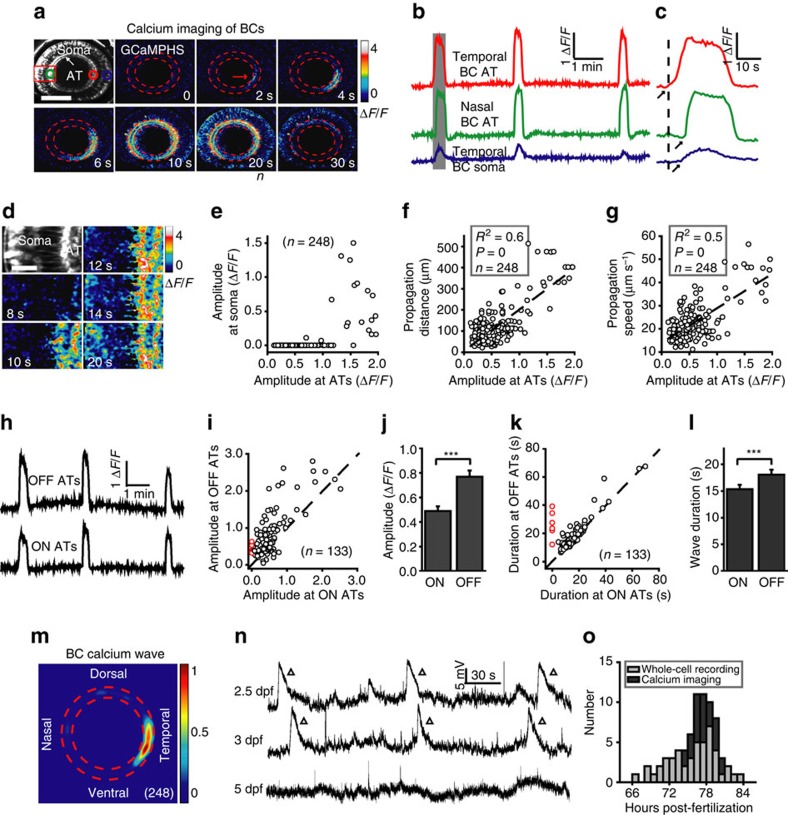
Developing bipolar cells display spontaneous wave activities in intact zebrafish larvae. (**a**) Pseudocolor time-lapse two-photon images showing a spontaneous calcium wave of bipolar cells (BCs) in a 3 d.p.f. Tg(Gal4-VP16^xfz43^,UAS:GCaMPHS) larva. Left top, fluorescence image showing the retinal structure and GCaMPHS expression in BCs. ‘AT' and ‘Soma' indicate the axon terminal and soma of BCs, respectively. Red arrow, initiation site of the wave. The area between the two red dashed circles is the location of the inner plexiform layer (IPL). Dorsal is up, and temporal is right. The same orientation is used for images in all of the following figures. Scale bar, 100 μm. (**b**) Calcium activities of three regions of interest in (**a**, colour circles). The shadowed area marks the period of images shown in **a**. (**c**) The first calcium wave in **b** at a higher time resolution. The arrows mark the onset of calcium activities. (**d**) Magnified time-lapse images of the boxed region in **a** showing the invasion of calcium waves from ATs to somata of BCs. Scale bar, 20 μm. (**e**–**g**) Plots of the peak amplitude of calcium waves at BC ATs in the initiation site versus their peak amplitude at BC somata (**e**) and propagation distance (**f**) and speed (**g**) at the IPL. The dotted lines represent linear regression. *N*=number of waves recorded. (**h**) Calcium activities of ON and OFF ATs at the initiation sites of the three waves in (**b**). (**i**,**k**) Plots of the peak amplitude (**i**) or duration (**k**) of BC calcium waves at ON ATs versus that at OFF ATs. The dotted lines represent the orthogonal. The red circles indicate six BC waves, which only initiated and propagated at OFF ATs in the nasal retina (Supplementary Fig. 2c,d). (**j**,**l**) Summary of data showing the peak amplitude (**i**) or duration (**k**) of calcium waves at BC ON or OFF ATs, respectively. (**m**) Superposition of the initiation site of 248 BC calcium waves from 23 larvae. Colour bar, normalized frequency of wave initiation. (**n**) Spontaneous electrical activities of BCs monitored with *in vivo* whole-cell recording in larvae at 2.5–5 days post-fertilization (d.p.f.). The triangles mark wave-like events of giant depolarizing potentials (GDPs). (**o**) Temporal distribution of BC wave occurrence during development. Whole-cell recording and calcium imaging data were obtained from 47 and 23 larvae, respectively. ***P<0.001; Statistical analysis was performed with ANOVA for the data in (**f**,**g**) and two-tailed paired Student's *t*-test for the data in (**j**,**l**). Data are represented as mean±s.e.m.

We monitored spontaneous neural activities among BC populations by performing *in vivo* time-lapse two-photon calcium imaging of double transgenic Tg(Gal4-VP16^xfz43^,UAS:GCaMPHS) or Tg(Gal4-VP16^xfz43^,UAS:GCaMP1.6) zebrafish larvae, in which BCs express the genetically encoded calcium indicator GCaMPHS or GCaMP1.6 (Supplementary Fig. 1a,b, and see also ref. 26, 27). In larvae at around 3 d.p.f. when RGCs just become light sensitive^28^, BCs displayed spontaneous calcium waves (23 out of 105 larvae), which always started from a cluster of BC axon terminals (ATs) and then propagated to nearby BC ATs at the IPL (Fig. 1a–c, Supplementary Fig. 2a,b and Supplementary Movies 1 and 2). Waves with a large peak amplitude at the initiation site could invade BC somata at the inner nuclear layer (Fig. 1d,e), and propagated far away along the IPL with a high speed (Fig. 1f,g; *n*=248 waves, *P*<0.001, analysis of variance (ANOVA)). Among the total of 248 waves, 17 of them extensively swept across the entire IPL (Fig. 1a; Supplementary Fig. 2a). For all waves, the mean propagation distance was 116.2±5.8 μm (mean±s.e.m., ranged from 21.4 to 514.0 μm), equal to 30.4±1.3% of the perimeter of the IPL (ranged from 5.9 to 100%). The mean speed of wavefront was 22.6±0.5 μm s^−1^ (ranged from 11.2 to 56.5 μm s^−1^).

Further analysis showed that in 127 out of 133 BC waves we analysed, BC ATs at both the sublaminae *a* and *b* of the IPL (that is, OFF and ON BC ATs) were usually simultaneously activated during wave activities (Fig. 1h). In the other 6 BC waves, calcium activities started and propagated only in the OFF ATs at the nasal retina (Fig. 1i,k; Supplementary Fig. 2c,d). OFF ATs often exhibited calcium waves with a larger amplitude and a longer duration than ON ATs (Fig. 1i-l). These results differed from a recent mice study, in which ON and OFF BCs were found to depolarize and hyperpolarize, respectively, during wave activity^23^. Furthermore, instead of the random initiation of retinal waves reported in previous *in vitro* studies^3^,^4^,^25^, individual waves in each retina were initiated preferentially from the same site (Supplementary Fig. 3). Among the total of 248 waves, the majority of them (175 out of 248) originated from BC ATs at the temporal retina (Fig. 1m). In the remaining 73 waves, 40, 30 and 3 of them originated from BC ATs at the dorsal, nasal and ventral retina, respectively.

Due to the limited sensitivity of GCaMPHS or GCaMP1.6 in detecting retinal waves^26^,^27^, we then performed *in vivo* whole-cell patch-clamp recording to confirm the robust existence of retinal waves in zebrafish larvae. In 47 out of 78 larvae at 2.5–3.5 d.p.f., 87 out of 142 BCs (including both ON and OFF BCs) exhibited spontaneous rhythmic giant depolarizing potentials (GDPs; Fig. 1n), which are a hallmark of retinal waves^3^,^4^,^28^. The earliest time point of BC wave occurrence was about 66 h post-fertilization (h.p.f.). Data obtained with *in vivo* whole-cell recording and calcium imaging together show that retinal waves in BCs usually occur between 66 and 84 h.p.f. (Fig. 1o). The mean interevent interval and event duration were 106.5±4.9 s and 10.6±0.3 s for GDPs (*n*=169), and 131.1±7.7 s and 21.5±0.6 s for calcium waves (*n*=248), respectively (Supplementary Fig. 4). The dynamic differences between GDPs and calcium waves of BCs may be due to the relative low sensitivity of calcium imaging and slow decay of intracellular calcium signalling^29^. These results indicate that BCs exhibit wave-like activities around 2.5–3.5 d.p.f., a period when the retina just possesses light responsiveness^28^.

### Axon terminals of BCs initiate retinal waves

**Figure 2:**
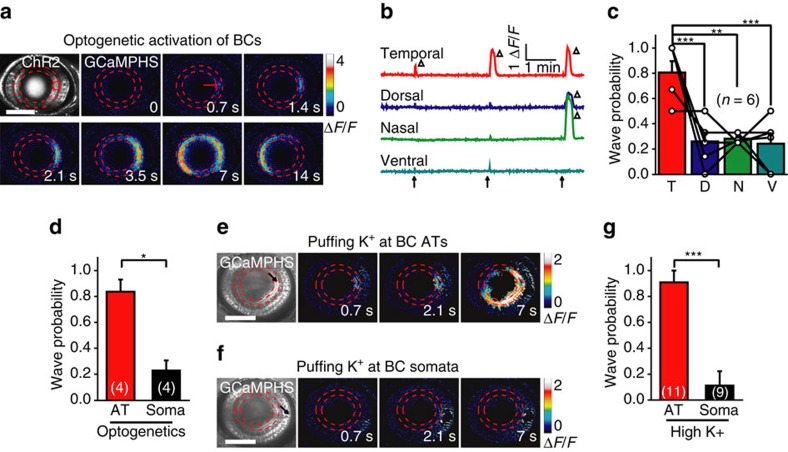
Axon terminals of BCs initiate retinal waves. (**a**) Time-lapse two-photon images showing a BC calcium wave evoked by 0.5 s optogenetic stimulation (started at time zero) at a cluster of ChR2-expressing BC ATs (red circle) in a transgenic Tg(Ribeye:ChR2-CFP,Gal4-VP16^xfz43^,UAS:GCaMPHS) larva. Left top, ChR2-CFP expression pattern in BCs. Red arrow, initiation site of the wave. Scale bar, 100 μm. (**b**) Repetitive optogenetic stimulation (arrow)-evoked calcium activities of BC ATs at four stimulation sites. The triangles mark the occurrence of calcium waves. (**c**) Occurrence probability of calcium waves evoked by optogenetics activation of BC ATs at different retinal regions. Data obtained from the same larva are connected by a line. The number in the brackets indicates the number of larvae examined. T, D, N and V indicate the temporal, dorsal, nasal and ventral retina, respectively. (**d**) Occurrence probability of calcium waves evoked by optogenetics stimulation at the AT or soma of BCs in the temporal retina. The number in the brackets represents the number of larvae examined. (**e**,**f**) BC calcium waves were evoked by local puffing of KCl (100 mM) at the ATs (**e**) but not the somata (**f**) in a 3-d.p.f. Tg(Gal4-VP16^xfz43^,UAS:GCaMP1.6) larva. The arrows in the bright-field images indicate the tip position of puffing micropipettes. The apparent increase in calcium signal around the puffing site (**f**) was due to puffing-induced displacement of BC somata. The puffing (0.1 s in duration) was performed at time zero. Scale bar, 100 μm. (**g**) Occurrence probability of calcium waves evoked by KCl puffing at the AT or soma of BCs in the temporal retina. *P<0.05, **P<0.01, ***P<0.001; one-way ANOVA for the data in (**c**), two-tailed paired Student's *t*-test for the data in **d**, two-tailed unpaired Student's *t*-test for the data in (**g**). Data are represented as mean±s.e.m.

As the calcium wave of BCs always started from the AT of BCs, we postulated that the BC AT is the initiation site of these waves. To address this question, we generated a triple transgenic zebrafish line Tg(Ribeye:ChR2-CFP,Gal4-VP16^xfz43^,UAS:GCaMPHS), in which ChR2 fused with cyan fluorescent protein (CFP) and GCaMPHS were co-expressed in BCs (Supplementary Fig. 5a,b). *In vivo* whole-cell recording showed that application of 440 nm laser stimuli evoked a fast and large depolarization in ChR2-expressing BCs (Supplementary Fig. 5c,d, in comparison with light-evoked responses in Supplementary Fig. 1c), indicating the optogenetic efficiency of ChR2 in BCs. Optogenetic activation of a small cluster of BC ATs within a circle (20 μm in diameter) by a 0.5-s pulse of 440-nm laser effectively triggered BC calcium waves, which started at the stimulation site and then propagated to nearby BC ATs along the IPL (Fig. 2a). Consistent with the preferential initiation of spontaneous BC waves at the temporal retina (Fig. 1m), optogenetic activation of BC ATs at the temporal retina was easier to trigger calcium waves than at the dorsal (*P*<0.001), nasal (*P*<0.01) or ventral retina (*P*<0.001) (Fig. 2b,c and Supplementary Movie 3; *n*=6 larvae, one-way ANOVA). Furthermore, at the temporal retina, calcium waves were more readily induced when optogenetic stimulation was applied to the ATs rather than somata of BCs (Fig. 2d; *n*=4 larvae, *P*<0.05, two-tailed paired Student's *t*-test). As a control, the same laser stimulation could not evoke calcium waves in Tg(Gal4-VP16^xfz43^,UAS:GCaMPHS) larvae (Supplementary Fig. 6). Consistently, BC waves could be induced when high KCl-containing solution (100 mM) was locally puffed to the ATs but not somata of BCs (Fig. 2e–g; P<0.001, two-tailed unpaired Student's *t*-test). These results indicate that the activation of BC ATs is sufficient to trigger the generation of retinal waves. Together with the fact that the calcium wave always stems from BC ATs and then back-propagates to BC somata (Fig. 1a–d), our findings suggest that the AT of BCs is the initiation site of retinal waves.

### Glutamate release from BC ATs is crucial for wave generation

**Figure 3:**
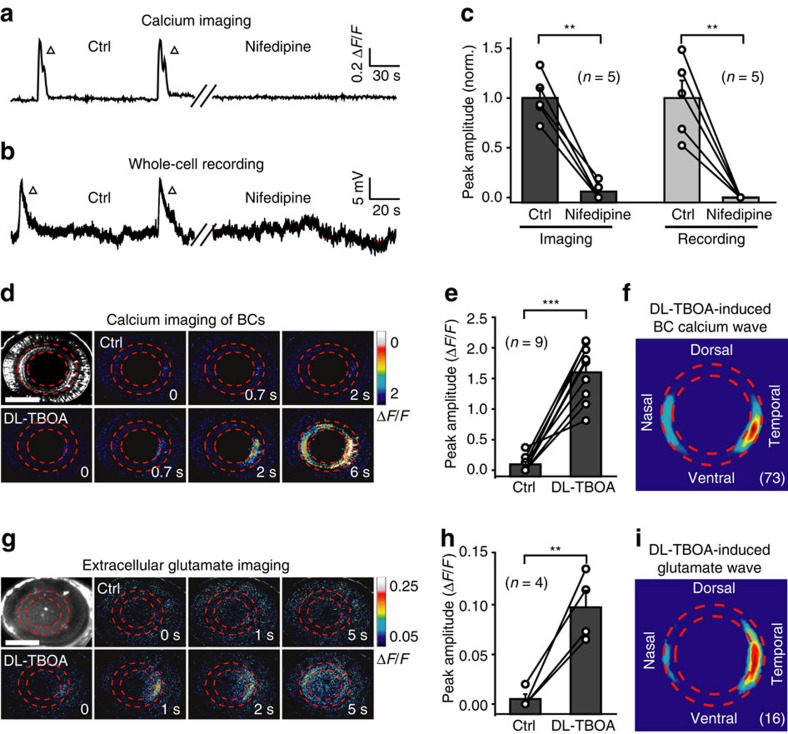
Glutamate release from BC ATs is important for BC wave occurrence. (**a**,**b**) Examples showing spontaneous calcium activities (**a**) and electrical activities (**b**) of BCs before (left) and 10 min after (right) bath application of nifedipine (50 μM). (**c**) Summary of data showing the effects of nifedipine on BC waves. Data obtained from the same larva are connected by a line. (**d**,**e**) Examples and summary of data showing that BC calcium waves were facilitated by bath application of DL-TBOA (50 μM) in a 3-d.p.f. Tg(Gal4-VP16^xfz43^,UAS:GCaMP1.6) larva. Scale bar, 100 μm. (**f**) Superposition of the initiation site of 73 BC calcium waves occurred under DL-TBOA application. (**g**) *In vivo* iGluSnFR-based extracellular glutamate imaging showing glutamate signals before (top) and 20 min after (bottom) bath application of DL-TBOA (50 μM) in a 3 d.p.f. larva expressing the glutamate biosensor iGluSnFR (left top). Scale bar, 100 μm. (**h**) Summary of glutamate imaging data. (**i**) Superposition of the initiation site of 16 glutamate waves under DL-TBOA application. **P<0.01, ***P<0.001; two-tailed paired Student's *t*-test for the data in (**c**,**e**,**h**). Data are represented as mean±s.e.m.

As the activation of BC ATs can trigger glutamate release, we next examined the role of glutamate release from BC ATs in wave generation. We applied nifedipine (50 μM) to block L-type calcium channels (LTCCs), which are densely located at BC ATs and critical for glutamate release from these terminals^30^,^31^, and found that both spontaneous calcium waves and GDPs of BCs were largely abolished (Fig. 3a-c; *P*<0.01, two-tailed paired Student's *t*-test). By contrast, increasing extracellular glutamate spillover by application of the glutamate transporter blocker DL-threo-beta-benzyloxyaspartate (DL-TBOA, 50 μM) markedly facilitated the generation of BC calcium waves in larvae, which displayed no or weak spontaneous activities under control condition (Fig. 3d,e; *P*<0.001, two-tailed paired Student's *t*-test). Similar to spontaneous waves (Fig. 1m), DL-TBOA-induced waves also originated mainly from BC ATs at the temporal retina (Fig. 3f; *n*=73). Inhibitory synaptic inputs from GABAergic and glycinergic amacrine cells (ACs) dynamically regulate glutamate release from BC ATs^30^,^32^,^33^. We found that, similar to DL-TBOA treatment, bath application of picrotoxin (200–500 μM), an antagonist of ionotropic GABA and glycine receptors, markedly enhanced BC calcium waves (Supplementary Fig. 7a,b; *P*<0.001, two-tailed paired Student's *t*-test). picrotoxin -induced waves also started preferentially from the temporal retina (Supplementary Fig. 7c; *n*=76). These findings suggest that glutamate release from BC ATs is important for the generation of BC waves.

We then performed *in vivo* glutamate imaging to visualize the spatiotemporal profile of extracellular endogenous glutamate in the intact zebrafish retina. The glutamate biosensor iGluSnFR (intensity-based glutamate-sensing fluorescent reporter) was non-selectively expressed in retinal cells by injecting its mRNA into embryos at one-cell stage (Methods)^34^. Although spontaneous wave-like glutamate signals were rare under control conditions possibly due to the low sensitivity and expression level of iGluSnFR, glutamate wave activities were observed within the IPL after blockade of glutamate uptake by DL-TBOA (50 μM; Fig. 3g,h). Interestingly, these extracellular endogenous glutamate waves also preferentially started at the temporal IPL (Fig. 3i; *n*=16). Considering the fact that BCs are the primary source of glutamate in the IPL^30^,^31^, these results further indicate the involvement of glutamate release from BC ATs in wave generation.

### NMDA receptors expressed at BC ATs mediate wave generation

**Figure 4:**
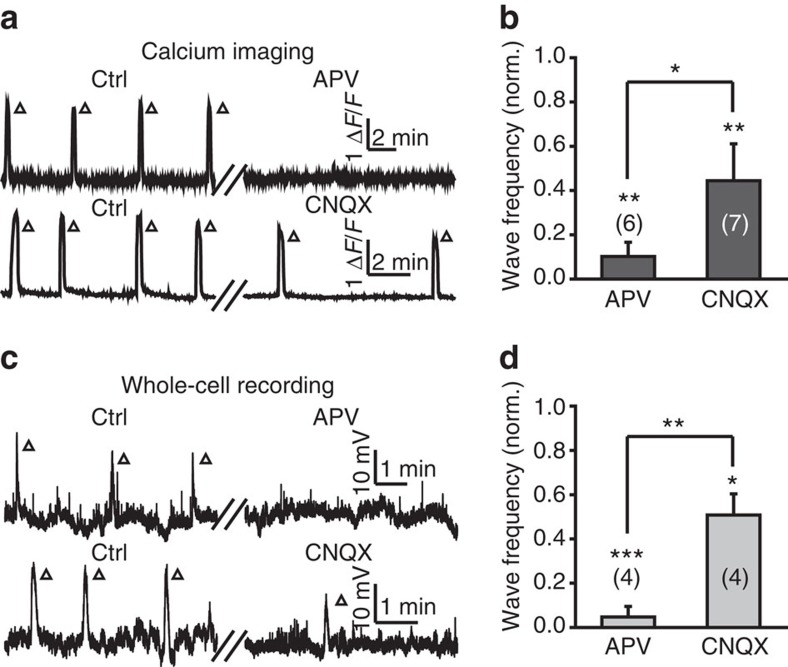
Ionotropic glutamate receptors are necessary for BC wave occurrence. (**a**,**c**) Effects of APV (top, 100 μM) and CNQX (bottom, 50 μM) bath application on spontaneous calcium waves (**a**) or GDPs (**c**) of BCs in 3-d.p.f. larvae. (**b**,**d**) Summary of calcium imaging (**b**) and whole-cell recording data (**d**). The number in the brackets represents the number of retinae imaged (**b**) or BCs recorded (**d**). *P<0.05, **P<0.01, ***P<0.001; two-tailed paired Student's *t*-test for the data between control and drug treatment, and two-tailed unpaired Student's *t*-test for the data between APV and CNQX. Data are represented as mean±s.e.m.

Considering the importance of glutamate release in the generation of BC waves, we hypothesized that BC ATs themselves may express presynaptic glutamate autoreceptors, which sense and boost spontaneous glutamate release and thus lead to the generation of BC waves. To examine this possibility, we first performed pharmacological experiments and found that both calcium waves and GDPs of BCs were abolished by the incubation of DL-2-amino-5-phosphonovaleric acid (APV, 100 μM), an antagonist of NMDARs, and partially suppressed by 6-cyano-7-nitroquinoxaline-2,3-dione (CNQX, 50 μM), an antagonist of the α-amino-3-hydroxy-5-methyl-4-isoxazolepropionic acid (AMPA) subtype of glutamate receptors (AMPARs; Fig. 4), indicating a critical role of ionotropic glutamate receptors, especially NMDARs, in the occurrence of glutamatergic waves in BCs.

**Figure 5:**
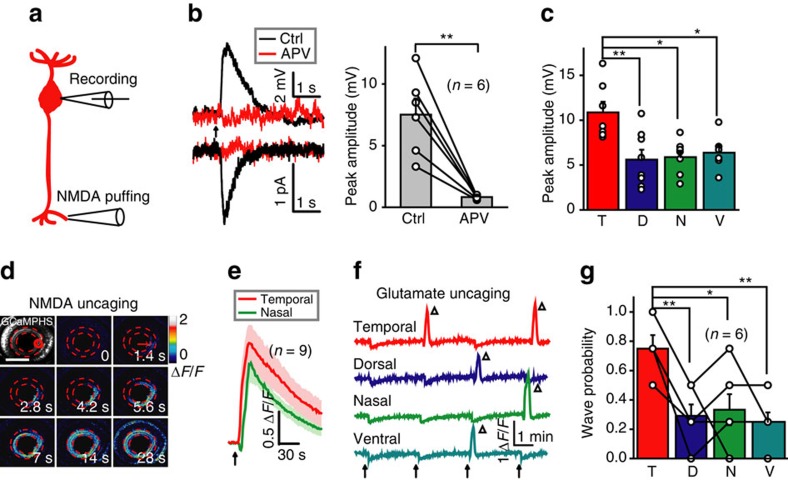
Activation of NMDARs at BC ATs is sufficient to initiate BC waves. (**a**) Schematic of simultaneous whole-cell patch-clamp recording at a BC soma and NMDA-containing solution puffing at a BC AT. (**b**) Left, NMDA puffing-evoked responses of BCs under current- (top) or voltage-clamp mode (bottom; holding potential, −60 mV) before (black) and after (red) application of APV (50 μM). The arrow marks the onset of puffing (0.1 s in duration). Right, summary of data. (**c**) Summary of NMDA-evoked responses of BCs in four different retinal regions. (**d**) BC calcium wave evoked by 0.5 s uncaging of NMDA (started at time zero) at a cluster of BC ATs in the temporal retina (red circle). Scale bar, 100 μm. (**e**) Mean calcium responses of temporal (uncaging site) and nasal BC ATs evoked by NMDA uncaging (arrow). The shadows represent error bars. (**f**) Repetitive glutamate uncaging (arrow)-evoked calcium activities of BC ATs at four uncaging sites. The triangles mark the occurrence of calcium waves. The slow reduction of calcium activities associated with each uncaging stimulus was due to laser-induced bleaching. (**g**) Occurrence probability of calcium waves evoked by glutamate uncaging at BC ATs in four different retinal regions. *P<0.05, **P<0.01; two-tailed paired Student's *t*-test for the data in **b**, one-way ANOVA for the data in **c** and **g**. Data are represented as mean±s.e.m.

We then performed *in vivo* whole-cell recording on BC somata in transgenic Tg(Gal4-VP16^xfz43^,UAS:nfsB-mCherry) larvae and locally puffed NMDA-containing solution (1 mM) to their ATs at the IPL (Fig. 5a). Under blockade of synaptic transmission by replacement of Ca^2+^ with Co^2+^ in the external solution, NMDA puffing evoked robust responses, which were totally suppressed by incubation of APV (Fig. 5b; *P*<0.01, two-tailed paired Student's *t*-test). Interestingly, NMDA application to BC ATs at the temporal retina evoked a larger response than at the dorsal (*P*<0.01), nasal (*P*<0.05) or ventral retina (*P*<0.05; Fig. 5c; one-way ANOVA), a phenomenon reminiscent of the preferential initiation of BC calcium and glutamate waves at the temporal retina (Figs 1m, 2c and 3f,i).

Furthermore, focal application of NMDA at a small cluster of BC ATs within a circle (20 μm in diameter) via 405-nm laser-based uncaging of MNI-caged-NMDA (10 mM; Methods) effectively induced calcium waves of BCs in all larvae examined (Fig. 5d,e; *n*=9). Similarly, focal uncaging of glutamate also evoked BC calcium waves, which were completely suppressed by APV application (Supplementary Fig. 8). Consistent with the effect of BC optogenetic activation (Fig. 2c), uncaging glutamate at the temporal retina was easier to induce BC calcium waves than at the dorsal (*P*<0.01), nasal (*P*<0.05) or ventral retina (*P*<0.01; Fig. 5f,g; *n*=6 larvae, one-way ANOVA). Taken together, these results indicate that BC ATs express functional NMDARs, which underlie the generation of BC glutamatergic waves. Furthermore, the intercellular asymmetry of NMDAR expression at BC ATs and resting membrane potentials of BCs (Supplementary Fig. 1d) at different retinal regions can account for the preferential initiation of BC waves at the temporal retina.

### Gap junctions between BCs are necessary for wave generation

**Figure 6:**
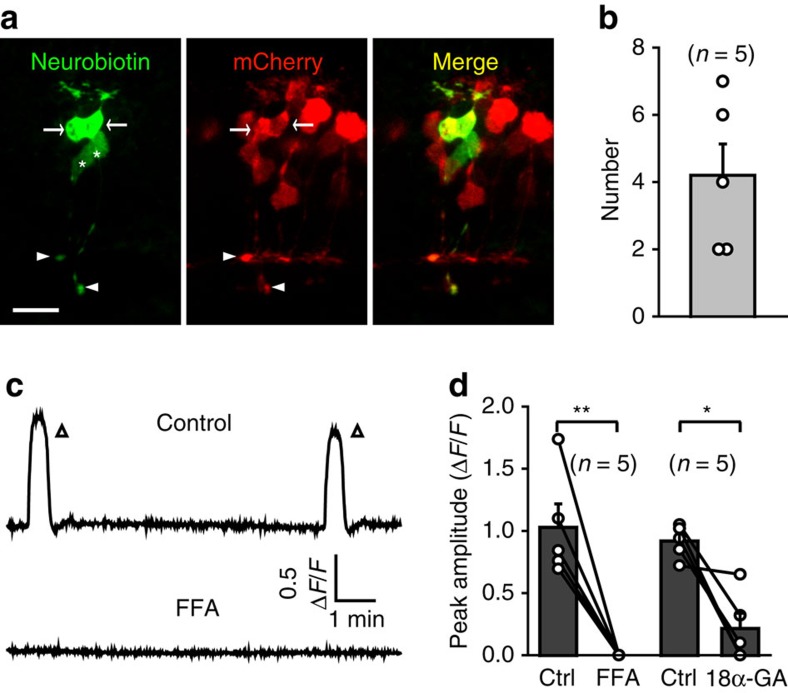
Gap junctions between BCs are necessary for the occurrence of BC waves. (**a**) Confocal images showing tracer coupling among nearby BCs. Neurobiotin was introduced into the BC soma of a 3-d.p.f. Tg(Gal4-VP16^xfz43^,UAS:nfsB-mCherry) larva through a recording micropipette. Due to the small size of zebrafish BCs, the soma of the recorded BC was pulled away by the removal of the micropipette. White arrows and arrowheads indicate the somata and ATs of coupled BCs. The white asterisks indicate two BCs with no expression of mCherry, because not all BCs express mCherry in Tg(Gal4-VP16^xfz43^,UAS:nfsB-mCherry) larvae. Scale bar, 10 μm. (**b**) Summary of data showing the number of coupled BCs. (**c**) Example showing spontaneous calcium activities of BCs before and 10 min after bath application of FFA (50 μM). (**d**) Summary of data showing the effects of FFA (50 μM) and 18α-GA (50 μM) on BC waves. Data obtained from the same larva are connected by a line. *P<0.05, **P<0.01; two-tailed paired Student's *t*-test for the data in **d**. Data are represented as mean±s.e.m.

Besides chemical synaptic neurotransmission, gap junctions have been implicated to facilitate the propagation of retinal waves^14^,^23^. To examine the role of gap junctions in BC waves of larval zebrafish, we first loaded 2.5% neurobiotin into the soma of BCs in larvae at 2.5–3.5 d.p.f. via whole-cell recording pipettes. By staining with streptavidin-conjugated FITC, we found that additional 4.2±0.9 BCs nearby could be visualized in 5 out of 8 cases (Fig. 6a,b), indicating the existence of gap junctions between zebrafish BCs. Furthermore, bath application of the gap junction blocker flufenamic acid (FFA, 50 μM) or 18α-glycyrhetinic acid (18α-GA, 50 μM) almost abolished spontaneous BC calcium waves (Fig. 6c,d). These results indicate that gap junctions between BCs are required for the generation and lateral propagation of BC glutamatergic waves.

### Retinal wave can propagate from BCs to RGCs and optic tectum

**Figure 7:**
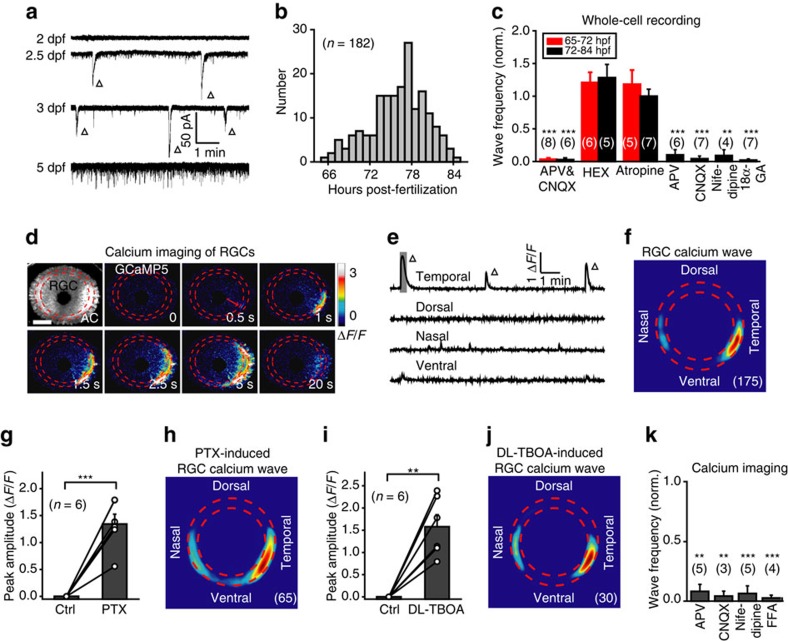
RGCs exhibit spontaneous retinal waves. (**a**) Spontaneous electrical activities of RGCs monitored with *in vivo* whole-cell recordings in 2- to 5-d.p.f. larvae. The triangles mark spontaneous wave-like GDPs. (**b**) Temporal distribution of the occurrence of spontaneous GDPs in RGCs (182 cells from 99 larvae). (**c**) Pharmacological properties of spontaneous GDPs in RGCs recorded during 65–72 h.p.f. (red) or 72–84 h.p.f. (black). The number in the brackets represents the number of RGCs recorded. APV&CNQX, 100 μM and 50 μM; HEX, 100 μM; atropine, 2 μM; APV, 100 μM; CNQX, 50 μM; nifedipine, 50 μM; 18α-GA, 50 μM. (**d**) Pseudocolor time-lapse two-photon images showing a spontaneous calcium wave of retinal ganglion cells (RGCs) in a 3 d.p.f. Tg(HuC:GCaMP5) larva. Left top, fluorescence image showing GCaMP5 expression in most of RGCs and some of amacrine cells (ACs). Red arrow, initiation site of the wave. Scale bar, 100 μm. (**e**) Calcium activities at four retinal regions. The shadowed area marks the period of images shown in **d**. (**f**) Superposition of the initiation site of 175 RGC calcium waves from 9 larvae. (**g**,**i**) Summary of the data showing the effect of picrotoxin (PTX, 200 μM) (**f**) or DL-TBOA (50 μM) (**h**) on RGC calcium waves. (**h**,**j**) Superposition of the initiation site of 65 RGC calcium waves induced by bath application of PTX (**g**) or 30 RGC waves by DL-TBOA (**i**). (**k**) Summary of the data showing the effects of APV (100 μM), CNQX (50 μM), nifedipine (50 μM) and FFA (50 μM) on the frequency of RGC calcium waves. The number in the brackets represents the number of retinae imaged. **P<0.01, ***P<0.001; two-tailed paired Student's *t*-test for the data in (**c**,**g**,**i**,**k**). Data are represented as mean±s.e.m.

As BCs are the primary excitatory synaptic source of RGCs and their ATs form glutamatergic synapses with RGC dendrites at the IPL^30^, it is of interest to examine whether retinal waves can propagate from BCs to RGCs. To address this question, we first performed *in vivo* whole-cell recording of RGCs from larvae aged 2–5 d.p.f. and found that RGCs displayed spontaneous periodic GDPs, which occurred between 65 and 84 h.p.f. (Fig. 7a,b; *n*=0/19 cells at 2.0–2.5 d.p.f.; *n*=30/50 at 2.5–3.0 d.p.f.; *n*=152/228 at 3.0–3.5 d.p.f.; *n*=0/92 at 3.5–6.0 d.p.f.), a time window similar to that of BC waves (see Fig. 1o). Regardless of when RGC waves occurred at early (2.5–3.0 d.p.f.) or late stages (3.0–3.5 d.p.f.), these activities were totally abolished by the co-application of APV (100 μM) and CNQX (50 μM) (Fig. 7c), but not the application of either hexamethonium (HEX, 100 μM) or atropine (2 μM), the antagonists of nicotinic and muscarinic acetylcholine receptors, respectively (Fig. 7c). It was noted that HEX treatment significantly reduced the amplitude of RGC waves (Supplementary Fig. 9). These results indicate that the occurrence of RGC waves in zebrafish is dependent on ionotropic glutamate receptors and modulated by nicotinic acetylcholine receptors.

We then performed two-photon calcium imaging of RGCs in 3-d.p.f. Tg(HuC:GCaMP5) larvae, in which the majority of RGCs and some of ACs expressed GCaMP5 (top left, Fig. 7d). As expected, RGCs also exhibited spontaneous calcium waves, which always started from the processes at the IPL and then propagated to the somata of some RGCs as well as ACs (Fig. 7d,e). Consistent with the initiation pattern of BC waves (Fig. 1m), these RGC waves were also preferentially initiated at the temporal retina (123 out of 175 waves from 9 retinae; Fig. 7e,f). Moreover, similar to BC waves, blockade of inhibition or glutamate transporters facilitated the occurrence of RGC waves in the retina, which originally displayed no or weak spontaneous activities under control conditions (Fig. 7g,i). Similar to spontaneous RGC waves (Fig. 7f), these pharmacological treatment-induced RGC waves also preferentially originated from the temporal retina (Fig. 7h,j; *n*=65 and 30, respectively).

Moreover, both calcium imaging and whole-cell recording showed that RGC waves were abolished after blockade of NMDARs by APV or blockade of AMPARs by CNQX (Fig. 7c,k; two-tailed paired Student's *t*-test). The stronger suppression effect of AMPAR blockade on RGC waves than on BC waves (comparison between Figs 4 and 7c,k) can be accounted for by the fact that synaptic transmission from BCs to RGCs is mainly mediated by postsynaptic AMPARs at RGC dendrites^27^,^30^. In addition, similar to BC waves, RGC waves were also abolished by blockade of LTCCs or gap junctions (Fig. 7c,k; two-tailed paired Student's *t*-test). Taken together, these results indicate that RGC waves share similar time course and pharmacological properties with BC waves.

**Figure 8:**
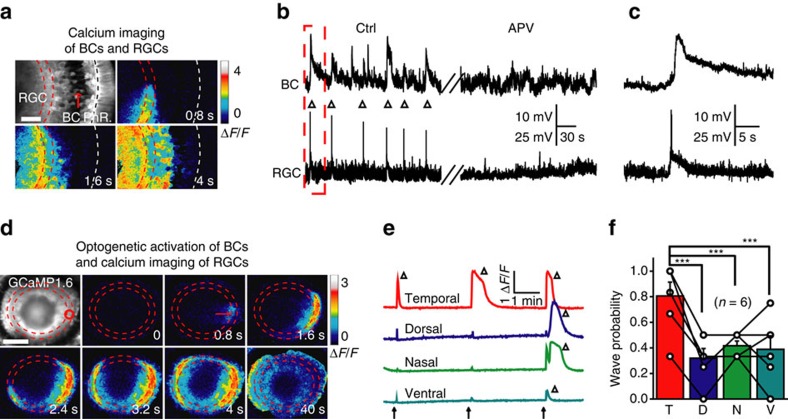
Glutamatergic waves can propagate from BCs to RGCs. (**a**) Calcium imaging of a 3 d.p.f. Tg(Gal4-VP16^xfz43^,Ath5:Gal4,UAS:GCaMP1.6) larva, showing that calcium waves initiated at the IPL can propagate to the soma of both BCs and RGCs. The region between the two red lines indicates the IPL and the white line marks the position of the outer plexiform layer (OPL). Scale bar, 20 μm. (**b**) *In vivo* dual whole-cell recording showing correlated spontaneous wave-like activities (triangles) between a BC and a RGC before (left) and after bath application of APV (right, 100 μM). (**c**) The first correlated events in **b** at a higher time resolution. (**d**) Time-lapse two-photon images showing a RGC calcium wave evoked by 0.5 s optogenetic stimulation (started at time zero) at a cluster of ChR2-expressing BC ATs (red circle) in a triple transgenic Tg(Ribeye:ChR2-CFP,Ath5:Gal4,UAS:GCaMP1.6) larva. Left top, GCaMP1.6 expression pattern in most of RGCs and some of ACs. Red arrow, initiation site of the wave. Scale bar, 100 μm. (**e**) RGC calcium activities evoked by repetitive optogenetic stimulation (arrows) of BC ATs at four different retinal regions. The triangles indicate the occurrence of RGC calcium waves. (**f**) Occurrence probability of RGC calcium waves evoked by optogenetics activation of BC ATs at four different retinal regions. ***P<0.001; one-way ANOVA for the data in **f**. Data are represented as mean±s.e.m.

We then simultaneously monitored spontaneous activities in both BCs and RGCs using calcium imaging of triple transgenic Tg(Gal4-VP16^xfz43^,Ath5:Gal4,UAS:GCaMP1.6) larvae, in which both BCs and RGCs express GCaMP1.6. Correlated spontaneous calcium waves were observed in these two types of cells. Waves were initiated at the IPL and then propagated to the somata of both BCs and RGCs (Fig. 8a). *In vivo* dual whole-cell recording of a BC and a RGC also showed that, corresponding to each spontaneous wave-like activity in the recorded RGC, there was always a correlated GDP in the BC (left, Fig. 8b,c). To directly examine whether wave activities can be transmitted from BCs to RGCs, we generated a triple transgenic line Tg(Ribeye:ChR2-CFP,Ath5:Gal4,UAS:GCaMP1.6), in which BCs and RGCs express ChR2 and GCaMP1.6, respectively. We found that optogenetic activation of a small cluster of BC ATs within a circle (20 μm in diameter) effectively induced RGC calcium waves, which started at the optogenetic stimulation site and propagated to the processes and somata of RGCs as well as ACs (Fig. 8d). Similar to the optogenetic effect on BC waves (Fig. 2b,c), optogenetic activation of BC ATs at the temporal retina was easier to trigger RGC waves than at the dorsal (*P*<0.001), nasal (*P*<0.001) or ventral retina (*P*<0.001) (Fig. 8e,f; *n*=6, one-way ANOVA). Considering that the optogenetic stimulation of BC ATs can induce BC waves, these findings together indicate that glutamatergic waves can propagate from BCs to RGCs.

**Figure 9:**
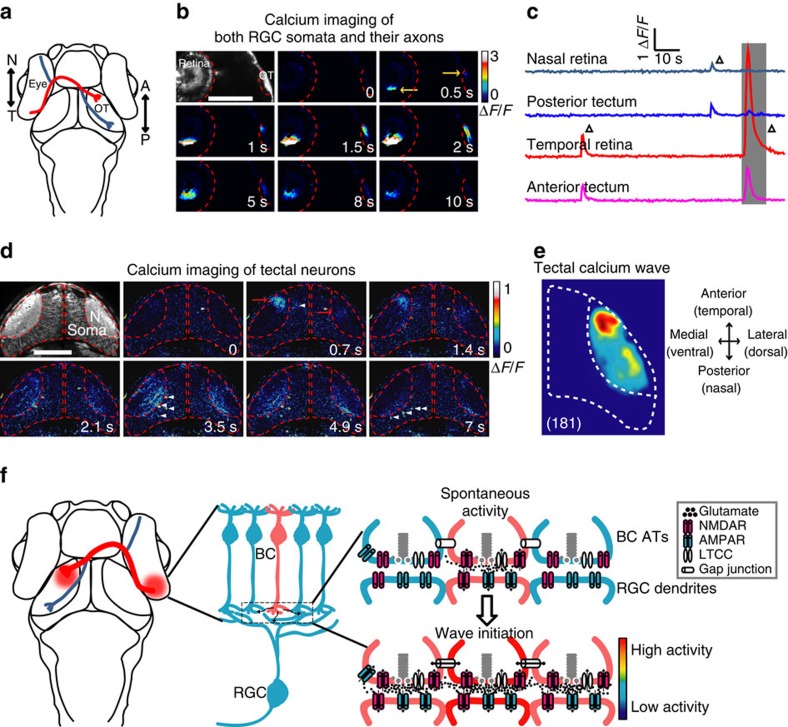
Retinal waves can propagate from RGCs to the optic tectum. (**a**) Schematic of the head of larval zebrafish and the projection of RGC axons from the eye to the optic tectum (OT). The red and blue lines indicate the axonal projections of RGCs at temporal (T) and nasal (N) retina into the anterior (A) and posterior (P) OT, respectively. (**b**) Pseudocolor time-lapse two-photon images showing correlated spontaneous calcium waves between RGC somata at the retina and their axon arborization at the OT of a 3 d.p.f. Tg(Ath5-gal4:UAS-GCaMP5) larva. Left top, fluorescence image showing GCaMP5 expression in RGCs and their axon terminals. Yellow arrows, initiation site of the waves in both retina and OT. Scale bar, 100 μm. (**c**) Calcium activities at two retinal and two tectal regions. The shadowed area marks the period of images shown in (**b**). (**d**) Pseudocolor time-lapse two-photon images showing spontaneous calcium waves in two OT hemispheres of a 3-d.p.f. Tg(HuC:GCaMP5) larva. Left top, fluorescence image showing GCaMP5 expression in the neuropil and soma layers of the OT. Red arrows, initiation site of the waves. White arrowheads, activated TNs during the propagation of the wave in the neuropil of the left OT. Scale bar, 100 μm. (**e**) Superposition of the initiation site of 181 calcium waves in the OT. Data were obtained from five larvae. (**f**) Schematic model showing the initiation of glutamatergic retinal waves. Spontaneously released glutamate from a BC (in pink, top) activates NMDARs at its own ATs. The activation of these presynaptic autoreceptors then depolarizes the membrane of ATs and consequently opens LTCCs, boosting glutamate release and leading to glutamate spillover within the IPL. Activation of NMDARs at neighbouring BC ATs by diffused glutamate and gap junctions between neighbouring BCs causes the lateral propagation of retinal waves at BC ATs along the IPL. On the other hand, glutamate also acts on postsynaptic AMPARs and NMDARs at the dendrites of RGCs, leading to the propagation of retinal waves from BCs to RGCs.

RGC axons projected topographically into the contralateral zebrafish OT, a similar structure to mammalian superior colliculus (Fig. 9a). To examine whether retinal waves can propagate to the OT, we first performed *in vivo* calcium imaging of both RGC somata in the retina and their axon arborization in the OT using Tg(Ath5:Gal4,UAS:GCaMP5) larvae. Correlated spontaneous calcium waves in the retina and OT were observed (Fig. 9b,c). Wave initiated at the temporal retina preferred to transmit to the anterior OT, while waves at the nasal retina to the posterior OT (Fig. 9b,c), indicating a topographical propagation of waves from the retina to the OT. Furthermore, we performed calcium imaging of the OT in 3-d.p.f. Tg(HuC:GCaMP5) larvae, in which the majority of RGCs and tectal neurons (TNs) express GCaMP5. Although we could not discriminate the RGC or TN identity of neuronal processes in the OT neuropil, we observed spontaneous calcium waves sweeping in the neuropil that preferentially initiated at the anterior region (Fig. 9d,e), consistent with the temporal preference of RGC wave initiation (Fig. 7f). Therefore, these findings together indicate that retinal waves can propagate to the OT.

## Discussion

It has been suggested that BCs provide excitatory inputs to RGCs to trigger glutamatergic retinal waves^4^,^22^,^23^. In the present study, we demonstrate that BCs themselves exhibit glutamatergic waves, which are initiated at their ATs via a NMDAR-dependent mechanism, and propagate to RGCs and the OT (Fig. 9f). Spontaneously released glutamate from BCs activates NMDARs expressed at their own ATs. The activation of these presynaptic autoreceptors can depolarize BC ATs and consequently open voltage-gated LTCCs, thus boosting more glutamate release from these ATs. AMPARs at BC ATs may also contribute to this process, though they are not indispensable. Extracellularly accumulated glutamate then can activate NMDARs at neighbouring BC ATs and both NMDARs and AMPARs at opposite RGC dendrites, leading to the generation and propagation of wave activities in the populations of BCs and RGCs. Gap junctions between adjacent BCs are also involved in this process.

As retinal waves have been observed in reptiles, birds and mammals but not in *Xenopus*^3^,^35^, it is guessed that retinal waves might be absent in species that rely on vision to capture preys and avoid predators early in the development of their visual systems in comparison with species, which grow up visually deprived inside a shell or womb^35^. This thought needs to be revisited, because our study found evidence for the existence of retinal waves in zebrafish larvae, which also require vision for prey capture and predator avoidance during developmental stages. Zebrafish develop very rapidly. In terms of its visual functions, the retina begins to be visually responsible at∼2.5 d.p.f. (ref. 28), RGCs begin to form synapses on TN dendrites at∼2 d.p.f. (refs 36, 37), and larvae are able to perform prey capture and predator avoidance behaviours from∼4–6 d.p.f. (ref. 38). Therefore, the occurrence of zebrafish retinal waves between 2.5–3.5 d.p.f. is coincident with the rapid development of retinal circuits and retinotopic maps^28^,^36^,^39^,^40^, but earlier than the appearance of prey capture and predator avoidance behaviours. Compared with the three distinct developmental stages of retinal waves in mammals, zebrafish retinal waves are totally dependent on ionotropic glutamatergic transmission and slightly modulated by nicotinic acetylcholinergic transmission. This difference may be caused by the advanced formation of functional BC-RGC glutamatergic synapses and the late development of cholinergic systems in zebrafish retinae^28^,^39^,^41^.

Previous pharmacological studies on *in vitro* retinal samples showed that the late-stage retinal waves could be blocked by the antagonists of ionotropic glutamate receptors, suggesting that BCs may be a source of excitatory drive for glutamatergic retinal waves^15^,^19^,^20^,^21^. The propagation of glutamatergic retinal waves was accompanied with extrasynaptic glutamate spillover around BC ATs^20^,^22^. Through dual patch-clamp recordings of a BC and a RGC in immature mouse retina, a recent study showed that BCs exhibited spontaneous periodic depolarization, which was correlated with the activity of RGCs, suggesting that BCs participate in glutamatergic retinal waves^23^. In the present study, using *in vivo* calcium imaging of zebrafish intact retinae, we found that BCs themselves display glutamatergic wave activities, which always stem from BC ATs. Furthermore, using optogenetics tools, we demonstrated that the axon terminal of BCs is the initiation site of glutamatergic retinal waves. In contrast to opposite participation of ON and OFF BCs in glutamatergic waves in mice^23^, both ON and OFF BCs or their ATs in zebrafish larvae were usually activated during waves, and OFF BC ATs often exhibited a stronger excitation than ON BC ATs. Due to the limited temporal resolution of calcium imaging, we could not distinguish which ATs of ON or OFF BCs were firstly activated.

Prolonged depolarization of BCs during wave activities will lead to long-lasting opening of LTCCs and cause a large amount of glutamate release into extracellular space^30^,^31^. Indeed, using iGluSnFR-based glutamate imaging^34^, we found that extracellular glutamate signalling propagates in a wave-like manner along the IPL. Its spatiotemporal pattern is similar to BC waves. Furthermore, blockade of glutamate release or elevation of glutamate spillover could, respectively, abolish or facilitate the occurrence of glutamatergic waves, indicating a critical role of extracellular glutamate in wave initiation. How does glutamate released from BC ATs mediate the generation of glutamatergic waves? Using *in vivo* whole-cell patch-clamp recordings of BCs, we found that BC ATs express functional NMDA autoreceptors. These receptors could be activated by spontaneously released glutamate, leading to depolarization of presynaptic membrane and boosting of vesicle release from BC ATs^42^,^43^. A large amount of released glutamate will accumulate and diffuse in synaptic and extrasynaptic regions along the IPL^20^,^22^, and then activate presynaptic NMDARs at neighbouring BC ATs, resulting in lateral propagation of wave activities among BC ATs. As expected, blockade or activation of NMDARs could abolish or induce wave generation, respectively (Figs 4 and 5d).

Extracellular glutamate profile at the IPL is tightly controlled by glutamate release and glutamate uptake^30^,^42^. The degree of glutamate release from BCs is dependent on the activation of BC ATs. As we found, the activation of NMDA autoreceptors could depolarize BC ATs and increase their glutamate release, leading to the facilitation of wave generation. In a recent study^24^, Ih channels at BC ATs were also found to play a positive role in the generation of glutamatergic retinal waves, possibly through its depolarization effect on BC ATs. On the other hand, ACs make inhibitory synapses on BC ATs and downregulate their glutamate release^32^,^33^. Glutamate transporters expressed at BC ATs and surrounding Müller glial processes can uptake excessive extracellular glutamate to restrain the concentration of extracellular glutamate^44^,^45^. Therefore, blockade of glutamate transporters or inhibition could enhance wave activities (Fig. 3d–f; Supplementary Fig. 7).

Most previous studies examined retinal waves in limited areas of isolated retinae^17^,^18^. Both cholinergic and glutamatergic retinal waves were found to be initiated randomly in different retinal sites and propagate with arbitrary directions^3^,^4^,^25^. However, our *in vivo* findings reveal that glutamatergic retinal waves display a stereotyped initiation pattern, as evidenced by the fact that they start preferentially at the temporal retina. Similarly, *in vivo* imaging of the superior colliculus of neonatal mice suggests that cholinergic retinal waves occur at the ventral–temporal retina and then propagate to the dorsal–nasal retina^5^,^8^,^12^. The discrepancy of wave initiation pattern between in vitro and *in vivo* studies is possibly due to the limited retinal regions used in studies *in vitro*^46^. For example, two *in vitro* studies on half of an isolated mouse retina showed that cholinergic waves exhibit a preferential propagation direction in nasal–temporal bias, and glutamatergic waves occur in a focused but not random pattern^47^,^48^. In the present *in vivo* study, we found that each region of the zebrafish retina is capable of generating glutamatergic retinal waves, but the threshold for wave generation is region-dependent (Figs 2c, 5g and 8f). Therefore, waves will certainly start from the retinal region with the lowest initiation threshold in intact retinae.

The stereotyped initiation of glutamatergic waves at the temporal retina was preserved after blockade of glutamate transporters or inhibitory receptors (Figs 3f and 7h,j; Supplementary Fig. 7c). Furthermore, optogenetic activation of BC ATs at the temporal retina could more easily trigger waves in both BCs or RGCs (Figs 2c and 8f). These findings suggest that the preference of wave initiation is not caused by non-uniform distribution of glutamate transporter or inhibitory receptors, but possibly due to a lower threshold for wave generation at temporal BC ATs. Consistent with this idea, we found that the BCs at the temporal retina exhibited a more depolarized resting membrane potential than those at other regions (Supplementary Fig. 1d). More importantly, the activation of NMDARs at the temporal retina causes the largest membrane depolarization of BCs and most easily evokes retinal waves (Figs 5c,g and 8f). Therefore, intercellular differences in NMDA autoreceptors at BC ATs and resting membrane potentials of BCs in different retinal regions may contribute to the preferential initiation of glutamatergic retinal waves.

The propagation of glutamatergic retinal waves along the IPL can cause repetitive synchronized activities between presynaptic BCs and postsynaptic RGCs. This can help to instruct the establishment of synaptic connections between synchronized BCs and RGCs via a Hebbian mechanism^27^,^49^. In the mouse retina, manipulation of spontaneous glutamate release from BCs markedly affected the rate of formation but not elimination of synapses formed by BC ATs on RGC dendrites^50^,^51^. Therefore, higher frequency of spontaneous wave activities at the temporal retina may contribute to the formation of more synaptic connections in this region, a phenomenon that has been observed in zebrafish and other teleosts^39^,^52^. Consistent with the findings in mice^6^,^7^,^8^, zebrafish retinal waves can propagate to the visual center optic tectum. Considering the rapid development of the visual system and the coincidence between retinal wave occurrence and visual development in zebrafish, the stereotyped wave initiation pattern may play an important role in guiding the refinement of retinofugal projections along the nasal–temporal axis, facilitating functional development of visual maps and providing higher visual acuity for frontal environment.

## Methods

### Zebrafish larval preparation

Adult zebrafish (*Danio rerio*) were maintained at the National Zebrafish Resources of China (Shanghai, China) with an automatic fish-housing system (ESEN, China) at 28 °C. Embryos and larvae were raised on a 14–10 h light–dark cycle in 10% Hank's solution, which consists of (in mM) 140 NaCl, 5.4 KCl, 0.25 Na_2_HPO_4_, 0.44 KH_2_PO_4_, 1.3 CaCl_2_, 1.0 MgSO_4_, 4.2 NaHCO_3_ (pH 7.2). Transgenic zebrafish lines used in this study include Tg(Gal4-VP16^xfz43^)^53^, Tg(Gal4-VP16^xfz43^,UAS:nfsB-mCherry)^53^, Tg(Ath5:Gal4)^54^, Tg(UAS:GCaMP1.6)^54^, Tg(UAS:GCaMPHS)^26^, Tg(UAS:GCaMP5), Tg(HuC:GCaMP5) and Tg(Ribeye:ChR2-CFP). In Tg(Gal4-VP16^xfz43^) fish, the majority of BCs express Gal4. In Tg(Ath5:Gal4) fish, the majority of RGCs, some of ACs and photoreceptors express Gal4. All *in vivo* time-lapse two-photon imaging and whole-cell recording were performed on 2- to 6-d.p.f. larvae at room temperature (22–26 ^o^C). For imaging experiments, 0.003% phenylthiourea (PTU) was added to the rearing solution to prevent pigmentation. All handling procedures were approved by the Institute of Neuroscience, Chinese Academy of Sciences.

### *In vivo* whole-cell patch-clamp recording

Zebrafish larvae were firstly paralysed with α-bungarotoxin (100 μg ml^−1^, Sigma), and then embedded in 1.2% low-melting-point agarose (Sigma) with one eye upwards in a custom-made chamber as described previously^27^,^28^. The cornea and lens of the upward eye were removed to expose retinal surface by using a glass micropipette with a tip opening of 1 μm. After the dissection, the larvae were transferred to a recording set-up, and perfused with extracellular solution, which consists of (in mM) 134 NaCl, 2.9 KCl, 4 CaCl_2_, 10 HEPES and 10 glucose (290 mOsmol l^−1^, pH 7.8). *In vivo* whole-cell patch-clamp recordings of BCs were performed on Tg(Gal4-VP16^xfz43^,UAS:nfsB-mCherry) larvae under fluorescent signal guidance, while recordings of RGCs were visualized under infrared illumination. Due to the small size of zebrafish BC somata, a recording micropipette with a tip opening of ∼1 μm was used for BC recording. Micropipettes were filled with (in mM) 100 K-gluconate, 10 KCl, 2 CaCl_2_, 2 Mg-ATP, 2 Na-GFP, 10 HEPES, and 10 EGTA (280 mOsmol l^−1^, pH 7.4). After the formation of giga-ohm seal, a brief negative pressure was applied to rupture the cell membrane beneath the micropipette tip. The equilibrium potential of Cl^−^ was about −60 mV according to the Nernst equation. Recordings were made with an EPC-10 amplifier (Heka, Germany), and signals were filtered at 2.9 kHz and sampled at 10 kHz. Due to the large input resistance of zebrafish BCs (7.3±0.9 GΩ, *n*=11), data were accepted if the series resistance was below 200 MΩ and varied <20% during the recording. For the recording of RGCs (input resistance: 3.3±0.5 GΩ, *n*=11), data were discarded if the series resistance was above 100 MΩ or varied >20%. Light-evoked responses of BCs were evoked by 2 s whole-field white flash controlled by an electrical shutter (LS6Z2, Uniblitz).

### *In vivo* two-photon calcium imaging

Calcium imaging experiments were carried out under a × 40 objective (numerical aperture (NA), 0.80) with an Olympus FV1000 confocal microscope (Olympus, Japan) equipped with a titanium: sapphire two-photon laser (Chameleon Ultra II, Coherent). The laser was tuned to 900 nm for exciting GCaMPs. For calcium imaging of BCs, larvae generated by crossing Tg(Gal4-VP16^xfz43^) with Tg(UAS:GCaMPHS) or Tg(UAS:GCaMP1.6) were used. For calcium imaging of RGCs and TNs, Tg(HuC-GCaMP5), Tg(Ath5:Gal4,UAS:GCaMP5) and Tg(Ath5:Gal4,UAS:GCaMP1.6) larvae were used. Imaging was performed on non-anaesthetized larvae, which were paralysed by α-bungarotoxin in some experiments to prevent muscle contraction. Time-lapse images with a resolution of 512 × 512 pixels were acquired at 1–2 Hz.

### Analysis of calcium-imaging data

Time-lapse images were processed by using custom-written programs in MATLAB (Mathworks) and ImageJ (NIH), and calcium waves were detected and analysed. For each time-lapse movie, the change in fluorescence intensity was calculated as Δ*F*/*F*=(F_*t*_−*F*)/*F*, where *F*_*t*_ was the fluorescence intensity at time *t*, and F was the average intensity of the entire movie. Each frame was filtered by smoothing the population activity with a 10-order Gaussian filtre. The onset of calcium waves was set if Δ*F*/*F* in the first frame was >5% of the peak amplitude of the wave during the raising phase^8^. Wave boundaries were defined as contiguous pixels that had Δ*F*/*F* >5% between the two consecutive frames^20^. The timing and threshold of each wave were confirmed based on visual inspection for better detection of wave boundaries. To exclude spontaneous calcium transients, we defined a continuous region larger than 200 μm^2^ (∼500 pixels) as an area threshold of a wave^17^. Calcium transients were excluded from further analysis.

The speed of wave propagation was calculated by dividing the propagation distance of the wavefront at the IPL by wave lifespan. The wavefront was determined by the wave boundary at each frame, and the propagation distance was measured by the furthest displacement of the wavefront. The measurement was confirmed by eye for each wave. Some weak waves were discarded if their wavefront was difficult to distinguish. To register the initiation site of all waves in a retinal template, the initiation site of each wave was first normalized according to its corresponding position at the IPL, then superimposed, and finally smoothed by a 10-order Gaussian filtre.

### *In vivo* iGluSnFR-based glutamate imaging

The iGluSnFR plasmid (Addgene, 41732) was linearized with PCiI restriction enzyme, and then used as a template for *in vitro* transcription into mRNA with mMESSAGE mMACHINE T7 kit (Ambion, USA). A dose of 3-ng iGluSnFR mRNA was pressure-injected into the animal pole of zebrafish embryos at the one-cell stage, and most of cells display green fluorescence signal at early development stages. Application of L-glutamate (1 mM) to iGluSnFR-expressing retinal cells could evoke an increase of fluorescence (Supplementary Fig. 10), indicating the efficiency of this glutamate biosensor. Due to gradual degradation of the mRNA, iGluSnFR fluorescence was very weak at 3-d.p.f. larvae. To reduce phototoxicity and bleaching, we performed *in vivo* glutamate imaging using a light-sheet Z.1 microscopy platform (Carl Zeiss, Germany) equipped with a highly sensitive sCMOS camera. Larvae were excited by one sheet of 488 nm light, which entered the eye from one side, and emitting fluorescent signals were collected with a × 20 Plan-Apochromat objective (NA, 1.0, Carl Zeiss) at the orthogonal direction. Time-lapse images with a resolution of 960 × 960 pixels were acquired at 5 Hz.

### ChR2-mediated optogenetic manipulation

To make Tol2-ribeye-ChR2-CFP plasmid, the promoter of zebrafish *ribeye a* was excised from a ribeye1.8 plasmid with NaeI and EcoRI restriction enzymes, and then cloned into the StuI and EcoRI sites of Tol2-UAS-ChR2-CFP plasmid. For the generation of Tg(Ribeye:ChR2-CFP) fish lines, the mixture of 25 pg DNA and 25 pg transposon mRNA was first injected into one-cell stage embryos, which were raised to adulthood for further screening of fluorescent signalling. F2 generation was used in optogenetic experiments. For optogenetic stimulation during calcium imaging, a 0.5 s pulse of 440-nm laser was applied to ChR2-expressing BC ATs within a circle (20 μm in diameter), while time-lapse images of calcium activities were simultaneously collected with a 900 nm or 488 nm laser via an Olympus FV1000 microscopy. In general, the volume of single BC ATs is about 3.14 × 2^2^ × 3 μm^3^. On the basis of the scattering of light in larval zebrafish brain's tissues^55^, it is speculated that around 50–100 BC ATs within a volume of 3.14 × 10^2^ × 10 μm^3^ (the depth of *z* axis is about 10 μm) were activated during the optogenetic activation.

### Laser-based uncaging of glutamate and NMDA

For uncaging experiments, larvae were incubated in the external solution containing MNI-caged-L-glutamate (10 mM; Tocris, UK) or MNI-caged-NMDA (10 mM; Tocris, UK) for 30 min after removal of eye lens. Focal photolysis of caged compounds was accomplished with a 405 nm laser, while time-lapse images of calcium activities were collected with a 488 nm laser via an Olympus FV1000 microscopy. A 0.5 s pulse of 405 nm laser was applied to the AT or soma of BCs within a circle (20 μm in diameter).

### Local puffing of drugs

For local application of drugs, a micropipette with a tip opening of ∼2 μm was placed at target areas. Drug-containing solution (NMDA, 1 mM; KCl, 100 mM) was ejected out by a brief air pressure (100 ms in duration, 10 psi in pressure), which was controlled by a Picospritzer III (Parker Instrumentation). Rhodamine dye was included in the internal solution to verify the reliability of puffing in each experiment.

### Gap junction tracing

Neurobiotin (2.5%) was loaded into the BC soma of Tg(Gal4-VP16^xfz43^,UAS:nfsB-mCherry) larvae via whole-cell patch-clamp recording micropipettes for >30 min. Due to the small size of zebrafish BCs, the soma of recorded cells was often pulled away by the removal of micropipettes. After the recording, the larvae were incubated in 4% paraformaldehyde (PFA) overnight at 4 ^o^C. At the next day, the larvae were first rinsed with 0.1 M PBST (PBS with 0.5% Tween) three times at the interval of 30 min, and then incubated in 0.1 M PBS containing 1:200 strepetavidin-conjugated FITC (Invitrogen) for 5 h at room temperature. After rinsed with 0.1 M PBST three times at the interval of 30 min, the tissue was embedded in 1.2% low melting-point agarose for the following imaging with Olympus FV1000 confocal microscope (excitation wavelength: 488 nm for FITC, 559 nm for mCherry).

### Statistical analysis

Lillietest function was first used to examine the normality distribution of data. For normal data of two-group comparison, two-tailed paired or unpaired student's *t*-test was used for significance analysis. Otherwise, Wilcoxen sign-rank test was used. For multiple-group comparisons, a one-way ANOVA test was first performed and then *post hoc* Bonferroni's multiple comparison test was used for comparisons between groups. The *P* value <0.05 was considered to be statistically significant. All results were represented as mean±s.e.m.

### Data availability

The data that support the findings of this study are available from the corresponding author on request.

## Additional information

**How to cite this article:** Zhang, R.-w. *et al*. Stereotyped initiation of retinal waves by bipolar cells via presynaptic NMDA autoreceptors. *Nat. Commun.* 7:12650 doi: 10.1038/ncomms12650 (2016).

## Supplementary Material

Supplementary Figures Supplementary Figures 1-10

Supplementary Movie 1 Consecutive spontaneous calcium waves of BCs in a 3-dpf Tg(Gal4-VP16xfz43,UAS:GCaMPHS) larva. The data was analyzed in Figure 1a-d. Replay speed: 30 frames/s.

Supplementary Movie 2 Multiple BC calcium waves with different spatial and temporal properties in a 3-dpf Tg(Gal4-VP16xfz43,UAS:GCaMP1.6) larva. The data was analyzed in Supplementary Figure 1d-f. Replay speed: 40 frames/s.

Supplementary Movie 3 BC calcium waves triggered by optogenetic activation of BC ATs in a Tg(Ribeye:ChR2-CFP,Gal4-VP16xfz43,UAS:GCaMPHS) larva. Three times of laser stimuli (white dots) were applied at each of four retinal regions. The data was analyzed in Figure 2a,b. Replay speed: 20 frames/s.
