# Supplementary material for: Stereotyped initiation of retinal waves by bipolar cells via presynaptic NMDA autoreceptors
Source: Nat Commun. 2016 Sep 2;7:12650. doi: 10.1038/ncomms12650 (PMC5025778; doi:10.1038/ncomms12650)
Supplement: Supplementary Figures — 1-10 [file ncomms12650-s1.pdf]

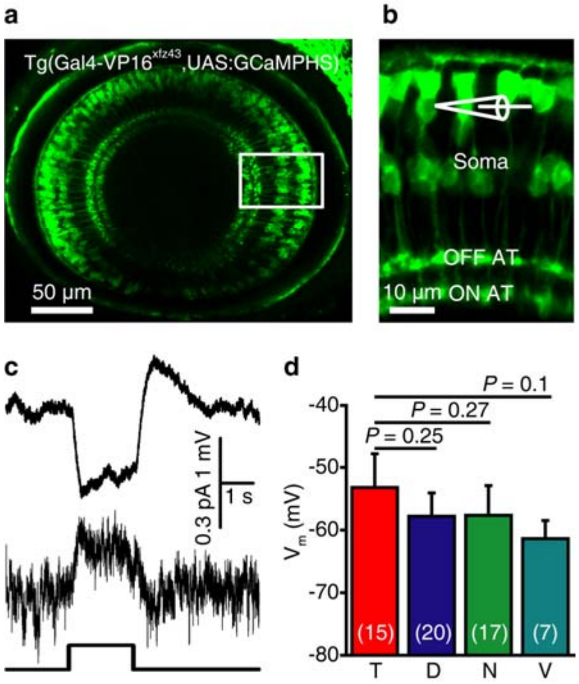

Supplementary Figure 1. Expression of GCaMPHS in BCs and electrophysiological properties of BCs in zebrafish larvae.

(**a**) Confocal image showing GCaMPHS expression in BCs of a 3-dpf Tg(Gal4-VP16^{xfz43},UAS:GCaMPHS) larva.

(**b**) Enlarged view of the boxed region in (**a**), showing the GCaMPHS expression in the soma, axon and AT of BCs. In vivo whole-cell recording was performed on BC somata.

(**c**) Light-evoked responses of an OFF subtype BC in response to a 2-s flash (bottom) under current- (top) or voltage-clamp mode (middle; holding potential, -60 mV).

(**d**) Summary of data showing the resting membrane potentials (V_m) of BCs at four different retinal regions. The number in the brackets indicates the number of BCs examined. T, D, N and V indicate the temporal, dorsal, nasal and ventral retina, respectively.

One-way ANOVA for the data in (**d**). Data are represented as mean ± s.e.m.

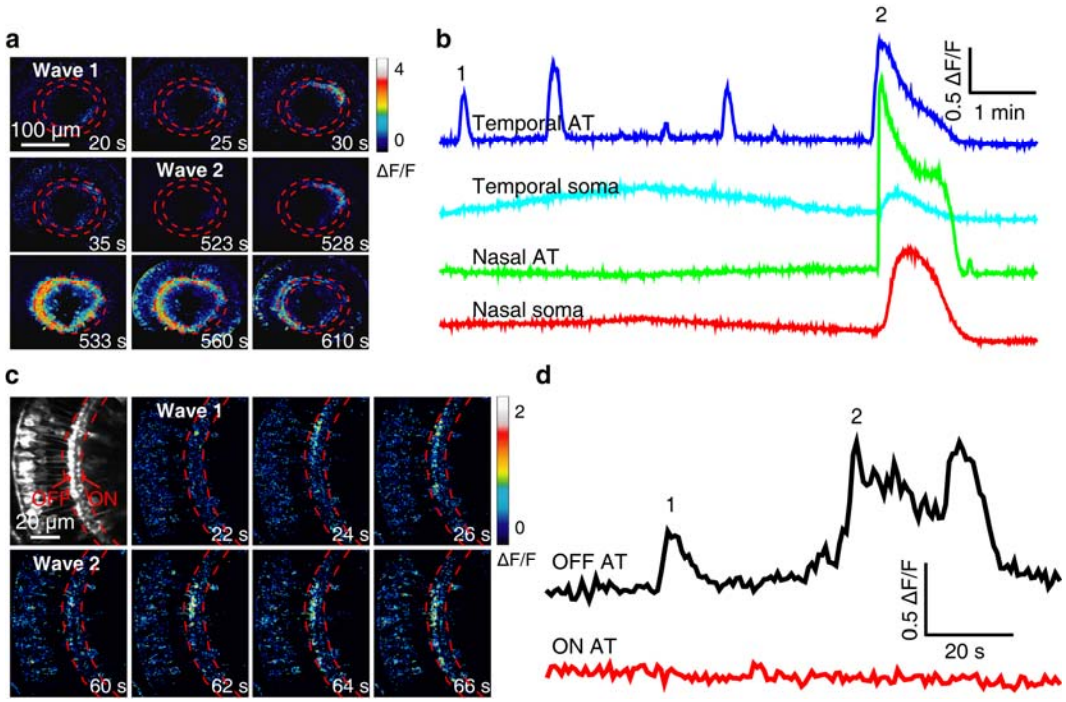

Supplementary Figure 2. Calcium wave activities in the soma and AT of BCs.

(**a**) Pseudocolor time-lapse images showing two BC calcium waves (1 and 2) in a 3-dpf Tg(Gal4-VP16^{xfz43},UAS:GCaMP1.6) larva. The wave 1 only propagated locally at BC ATs in the temporal-dorsal retina, while the wave 2 propagated globally to the entire IPL and evaded to BC somata.

(**b**) Spontaneous BC calcium activities at four different retinal regions. BC ATs at the temporal retina displayed several wave-like events. The first and last waves are showed in (**a**).

(c) Pseudocolor time-lapse images showing two BC calcium waves (1 and 2) in a 3-dpf Tg(Gal4-VP16^{xfz43},UAS:GCaMP1.6) larva. Both the two waves started and propagated locally at OFF ATs in the nasal retina. Left top, fluorescence image showing OFF and ON ATs which are located at the sublaminae *a* and *b* of the IPL, respectively.

(**d**) Spontaneous BC calcium activities at OFF ATs, but not ON ATs in the nasal retina. Corresponding images are showed in (**c**).

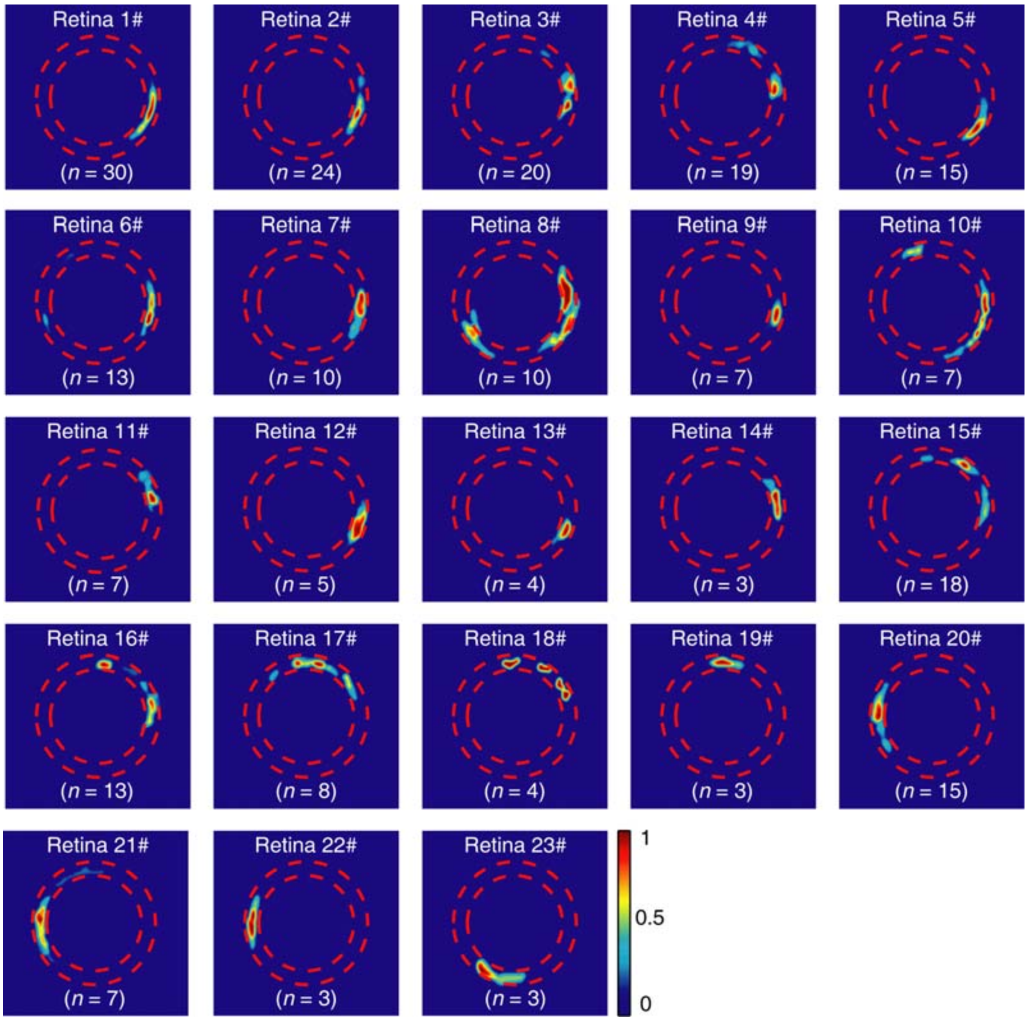

Supplementary Figure 3. Stereotyped initiation sites of multiple BC calcium waves in individual retinae.

The initiation site of all waves observed in individual retinae (n = 23) were superposed in heat maps. Waves in 14 retinae (#1 – #14) started mainly from BC ATs at the temporal retina, waves in 5 retinae (#15 – #19) from the dorsal region, waves in 3 retinae (#20 – #22) from the nasal region, and waves in 1 retina (#23) from the ventral region, though waves were initiated from 2 – 3 sites in some retinae (# 4, 8, 10, 15, 16, 17, 18). The number at bottom indicates the number of retinal waves occurred in each retina.

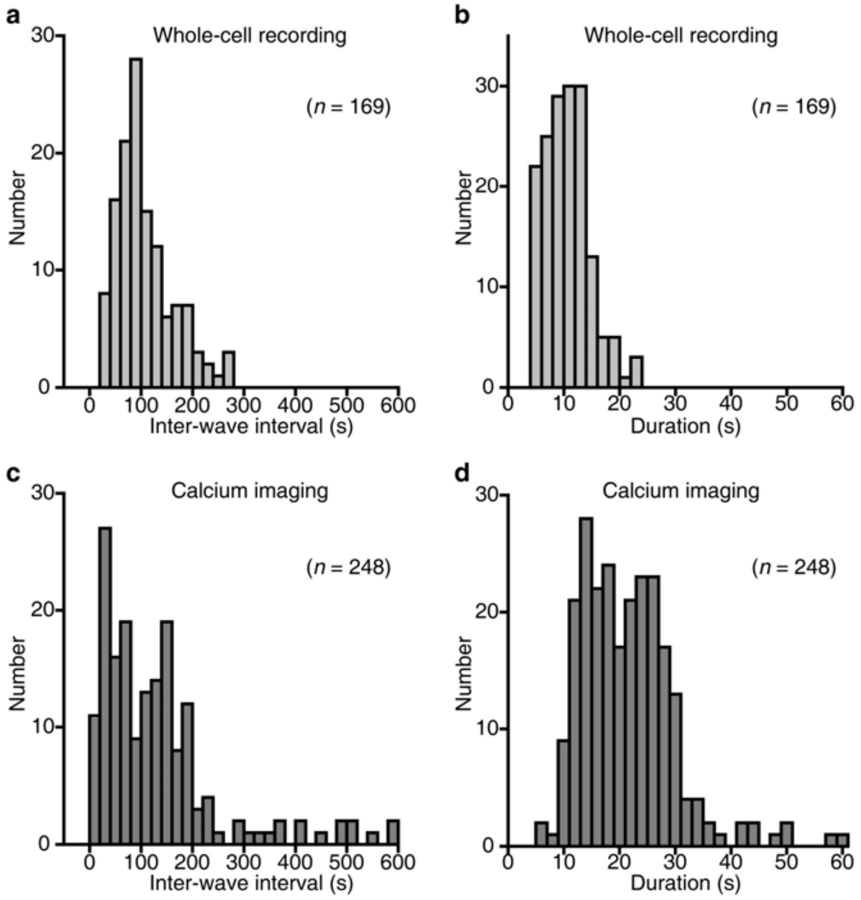

Supplementary Figure 4. Dynamic properties of BC waves.

(**a** and **b**) Distribution of the inter-wave interval (**a**) and duration (**b**) of spontaneous BC GDPs monitored with whole-cell recording in 3-dpf larvae. Data were obtained from 169 events in 33 BCs.

(**c** and **d**) Distribution of inter-wave interval (**c**) and duration (**d**) of spontaneous BC calcium waves monitored with calcium imaging in 3-dpf larvae. Data were obtained from 248 calcium waves in 23 retinae.

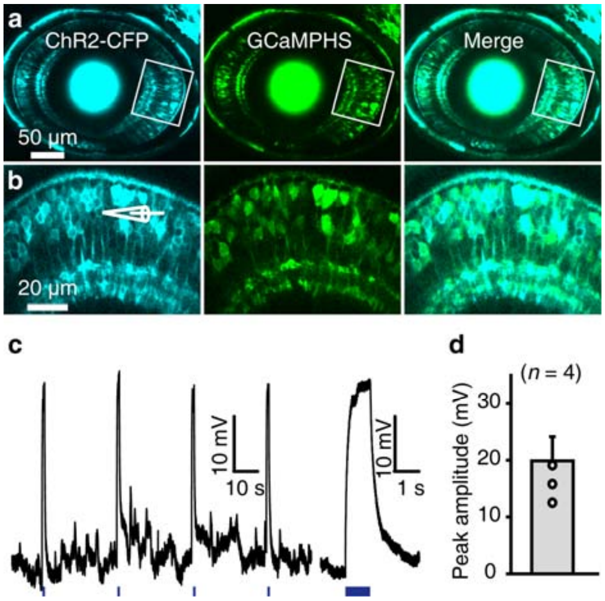

Supplementary Figure 5. Co-expression of ChR2-CFP and GCaMPHS in BCs.

(**a**) Confocal images showing the co-expression of ChR2-CFP and GCaMPHS in BCs of a 3-dpf Tg(Ribeye:ChR2-CFP,Gal4-VP16^{xfz43},UAS:GCaMPHS) larva.

(**b**) Enlarged view of the boxed region in (**a**).

(**c**) Depolarization of BCs in response to optogenetic stimulation (1 s in duration, 440 nm in wavelength, marked with blue lines at bottom). Right, the first depolarization response at a higher time resolution.

(**d**) Summary of optogenetic activation-induced BC responses, which are much larger than light-evoked BC responses (see Supplementary Figure 1c). Data are represented as mean ± s.e.m.

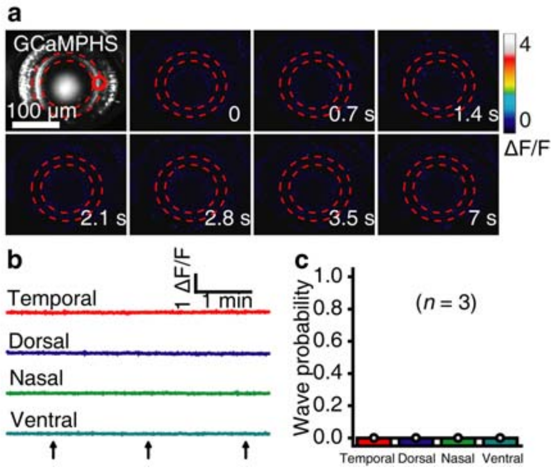

Supplementary Figure 6. ChR2 expression is required for optogenetic activation.

(**a-c**) Requirement of ChR2 expression for optogenetic activation. The same stimulation (started at time zero) on ChR2 non-expressing Tg(Gal4-VP16^{xfz43},UAS:GCaMPHS) larva did not evoke obvious calcium activity in BCs. Data are represented as mean ± s.e.m.

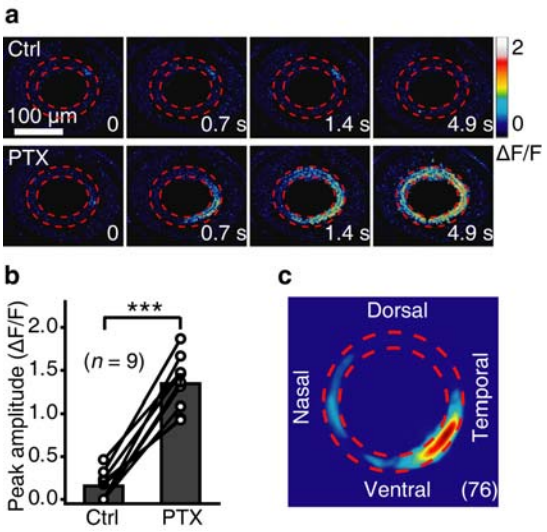

Supplementary Figure 7. Blockade of inhibitory inputs facilitates the occurrence of BC waves.

(**a**) Pseudocolor time-lapse images showing BC calcium activities before (top) and 20 min after bath application of picrotoxin (PTX, 200 μM) in a 3-dpf Tg(Gal4-VP16^{xfz43},UAS:GCaMP1.6) larva, which only exhibited local spontaneous calcium activities under control condition.

(**b**) Summary of data obtained from 9 larvae.

(**c**) Superposition of the initiation site of 76 BC calcium waves occurred under bath application of PTX.

***$P < 0.001$; two-tailed paired Student's t-test. Data are represented as mean ± s.e.m.

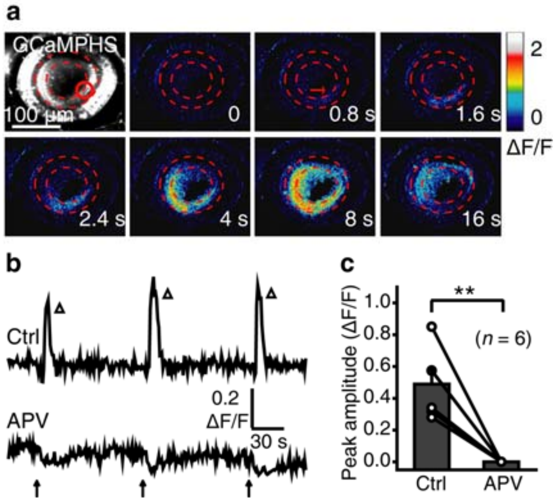

Supplementary Figure 8. Glutamate uncaging at BC ATs evokes NMDAR-dependent BC calcium waves.

(**a**) BC calcium wave evoked by 0.5-s uncaging of glutamate (started at time zero) at a cluster of BC ATs in the temporal-ventral retina (red circle) of a 3-dpf Tg(Gal4-VP16^{xfz43},UAS:GCaMP1.6) larva. Scale, 100 µm.

(**b** and **c**) Example (**b**) and summary (**c**) showing the effect of APV application (100 µM) on glutamate uncaging-induced BC calcium waves.

***P* < 0.01; two-tailed paired Student's t-test. Data are represented as mean ± s.e.m.

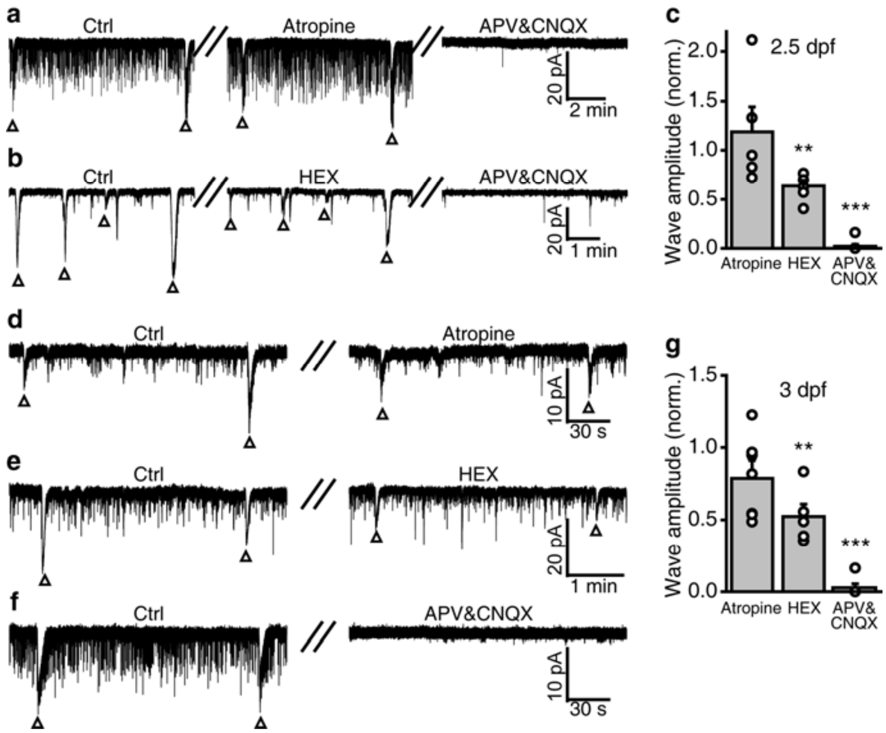

Supplementary Figure 9. Ionotropic glutamate receptors but not acetylcholine receptors are necessary for the occurrence of RGC waves.

(**a** and **b**) Examples showing spontaneous electrical activities of RGCs in 2.5-dpf larvae before (Ctrl) and after sequential bath-application of atropine (2 μM) and APV&CNQX (100 μM and 50 μM), or HEX (100 μM) and APV&CNQX (100 μM and 50 μM). The triangles mark the occurrence of GDPs.

(**c**) Summary of data showing the effects of atropine, HEX and APV&CNQX on the amplitude of RGC waves in 2.5-dpf larvae (n = 5, 6 and 8, respectively).The open circles indicate each of RGCs recorded.

(**d-f**) Examples showing spontaneous electrical activities of RGCs in 3-dpf larvae before and after bath-application of atropine (2 μM), HEX (100 μM), or APV&CNQX (100 μM and 50μM).

(**g**) Summary of data showing the effects of atropine, HEX and APV&CNQX on the amplitude of RGC waves in 3.0-dpf larvae (n = 7, 5 and 6, respectively). The open circles indicate each of RGCs recorded.

$^{**}P < 0.01$, $^{***}P < 0.001$; two-tailed paired Student's *t*-test for the data between control and drug treatment. Data are represented as mean ± s.e.m.

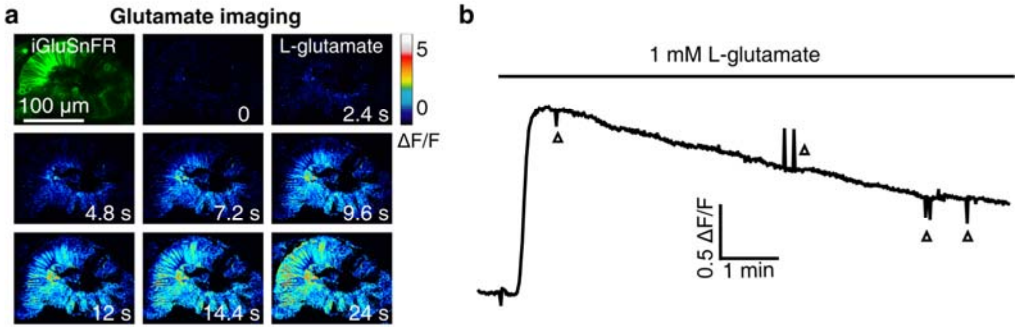

Supplementary Figure 10. Application of L-glutamate induces an increase of glutamate signal in iGluSnFR-expressing retinal cells.

(**a**) Pseudocolor time-lapse images showing the change of glutamate signal before and after bath application of 1-mM L-glutamate in the retina of a 44-hpf larva expressing the glutamate biosensor iGluSnFR (Left top). The lens of the retina was removed for the diffusion of L-glutamate into the retina.

(**b**) Time course of glutamate signal changes in the retina (**a**). The downward and upward deflections (triangles) were due to the movement of the larva during imaging.
